# Intrauterine Adhesion-Induced Septated Amniotic Cavity: Ultrasonographic Findings in Second and Third Trimesters

**DOI:** 10.3390/diagnostics14242826

**Published:** 2024-12-16

**Authors:** Jo-Ting Huang, Yu-Ming Chen, Ching-Chang Tsai, Hsin-Hsin Cheng, Yun-Ju Lai, Pei-Fang Lee, Te-Yao Hsu, Kun-Long Huang

**Affiliations:** 1 Department of Obstetrics and Gynecology, Kaohsiung Chang Gung Memorial Hospital and Chang Gung University Collage of Medicine, Kaohsiung 833401, Taiwan; tiffany100026@gmail.com (J.-T.H.); aniki@cloud.cgmh.org.tw (C.-C.T.); chokovarous@cloud.cgmh.org.tw (H.-H.C.); lusionbear@cloud.cgmh.org.tw (Y.-J.L.); pf7938@cgmh.org.tw (P.-F.L.); tyhsu@cgmh.org.tw (T.-Y.H.); 2 Department of Psychiatry, Kaohsiung Chang Gung Memorial Hospital and Chang Gung University Collage of Medicine, Kaohsiung 833401, Taiwan; yuming0320@gmail.com

**Keywords:** surgical evacuation, intrauterine adhesion, adhesive septum, cesarean section, uterine pseudocyst, Asherman syndrome

## Abstract

A 40-year-old woman who had obstetric history of one vaginal delivery and two surgical abortions to terminate early pregnancy received regular prenatal care without any systemic maternal diseases. During the detailed second trimester ultrasound, a homogenous adhesion-induced pseudocystic lesion of 8.6 × 7.4 cm was found between the inlet of the endocervix and the uterine cavity in the lower segment of the uterus. There was a clear septum with an inlet of about 2.6 cm near the right lower segment of the uterus. Transvaginal sonography showed a cervical length of 3.29 cm without dilatation. No gross fetal anomalies were found. Sometimes, the fetal head or limbs moved into this cystic space. At 36 3/7 weeks of gestation, a cesarean section was arranged for fetal breech presentation and pre-labor rupture of the membrane. After the delivery of the baby and its placenta, there was no obvious septum in the uterine cavity but only a very short fibrous tissue from the posterior wall of uterus, which could be destroyed when the baby was delivered. No adverse outcomes for the mother or the neonate were observed.

**Figure 1:**
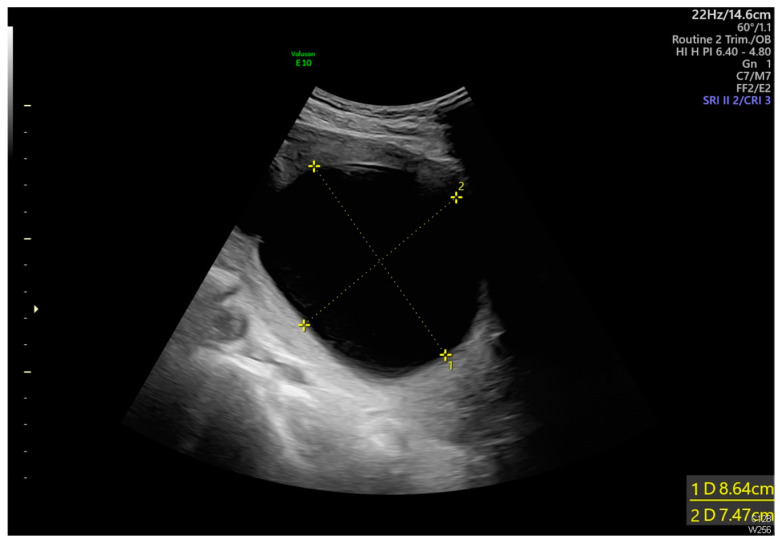
Transabdominal ultrasound revealed an adhesion-induced pseudocyst measuring 8.6 × 7.4 cm located between the endocervical inlet and uterine cavity in the lower uterine segment (As shown by the dotted line distance). The inlet was identified at the 10 o’clock position of the pseudocyst. The longest inlet of the adhesion-induced pseudocyst measured 2.6 cm (Figure 2). Uterine septa were present on anterior and posterior walls, resulting from intrauterine adhesions (Figure 3 and Figure 4). Transvaginal ultrasound showed cervical length of 3.29 cm without dilation, excluding cervical incompetence (CI) (Figure 5). Detailed second-trimester ultrasound detected no gross fetal anomalies or bulging uterine diverticulum. Fetal limbs and head moved freely into and out of the adhesion-induced pseudocavity without hyperechoic band-like lesions, making amniotic band syndrome unlikely. At 36 3/7 weeks’ gestation, cesarean delivery was performed due to pre-labor rupture of membranes and fetal breech presentation. Post-placental delivery examination revealed only a short fibrous tissue extending from the posterior uterine wall (Figure 6). The adhesion-induced septa may have been disrupted during fetal delivery. No adverse maternal or neonatal outcomes were observed.

**Figure 2:**
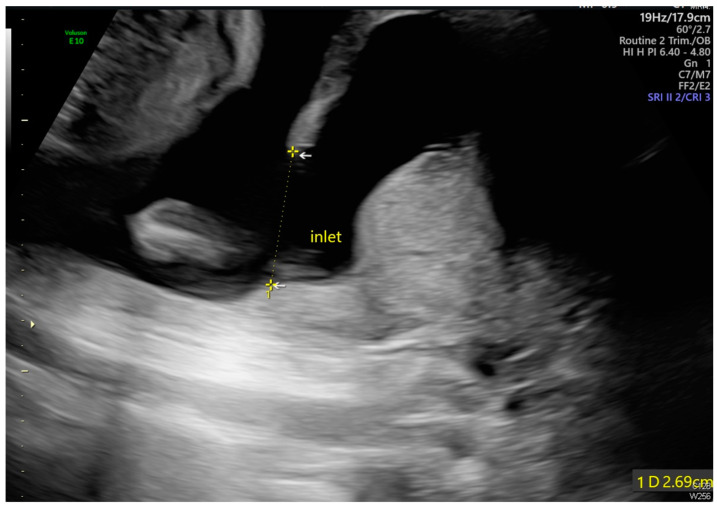
The maximal diameter of the pseudocyst’s opening measured 2.69 cm (As shown by the dotted line distance).

**Figure 3:**
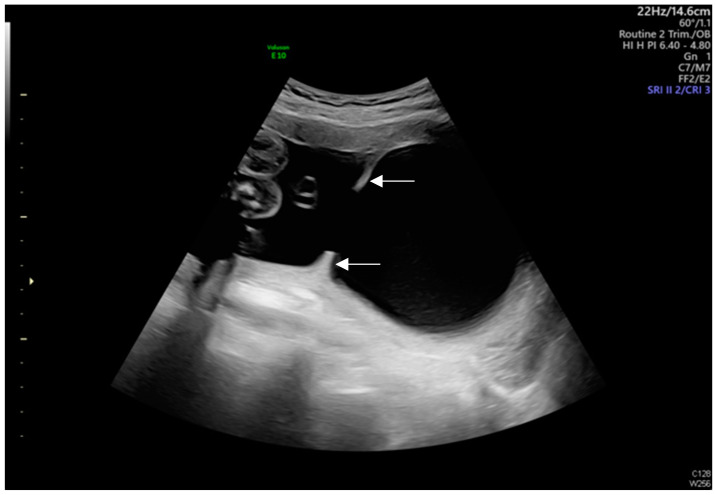
Uterine septa were present on anterior and posterior walls, resulting from intrauterine adhesions (As shown by the white arrows).

**Figure 4:**
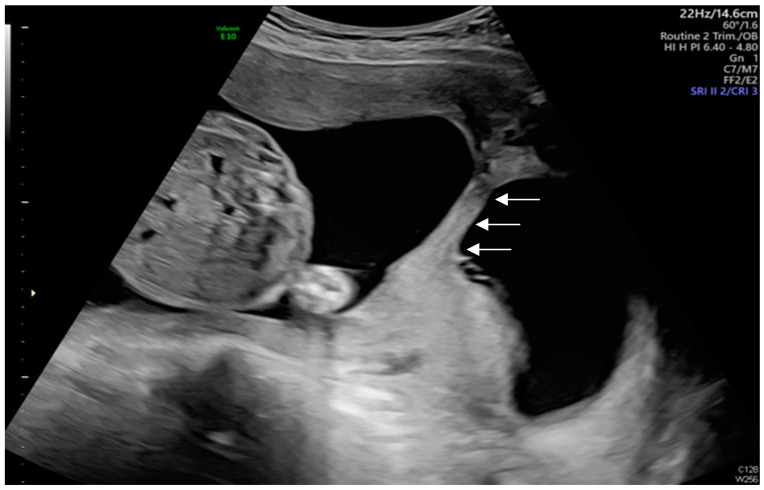
When the ultrasound probe was moved to the right side of the uterus, the adhesion-induced septa were visualized perpendicular to the anterior and posterior uterine walls, without inlet (As shown by the white arrows).

**Figure 5:**
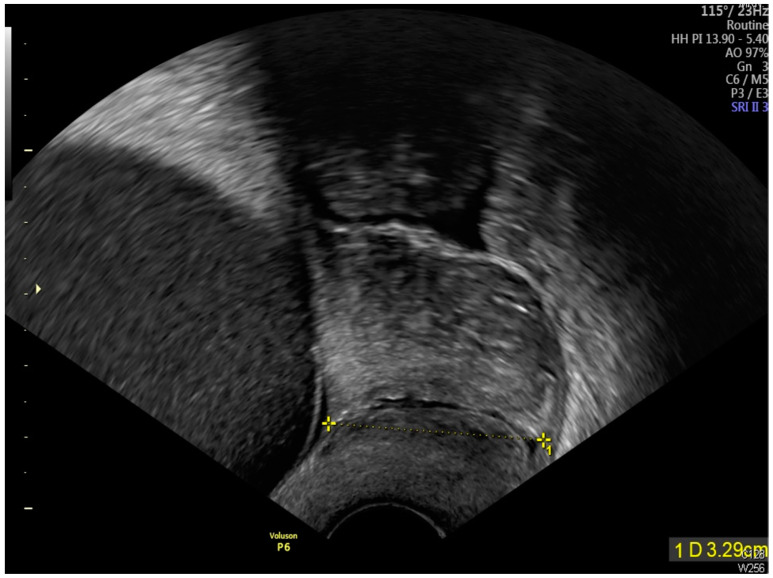
Transvaginal sonographic assessment demonstrated a cervical length measurement of 3.29 cm (As shown by the dotted line distance), with no evidence of cervical dilation.

**Figure 6:**
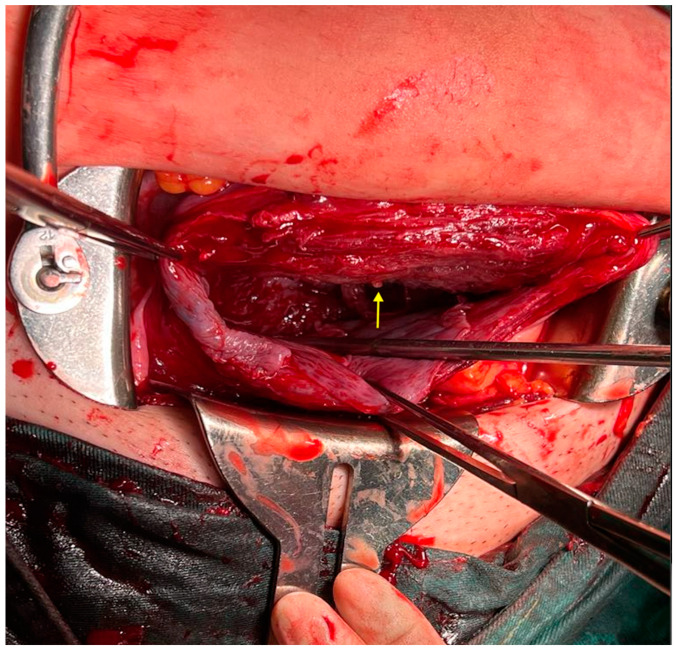
Examination after placental delivery revealed only a small strand of fibrous tissue extending from the posterior uterine wall (As shown by the yellow arrow). Dilation and curettage (D&C) is a common surgical procedure for managing pathogenic gestational tissue or elective pregnancy termination. In non-pregnant patients, it also serves as a therapeutic and diagnostic tool for abnormal uterine bleeding [1,2,3]. Although D&C is generally considered a safe procedure, it carries potential complications including cervical or uterine bleeding, infection, uterine perforation, and intrauterine adhesions [4,5,6]. Notably, injury to the decidua basalis leading to endometrial fibrous scarring and fusion of opposing surfaces is believed to be a possible mechanism for intrauterine adhesion formation [7]. Studies have also shown that recurrent pregnancy loss is associated with intrauterine adhesions, with evidence suggesting that hysteroscopic surgery may improve pregnancy outcomes [8].This series of images warrants discussion regarding the distinction among intrauterine adhesions, uterine diverticulum (UD), and CI during pregnancy. UD is an uncommon condition that may be either secondary to intervention/trauma or primarily developed [9]. It is usually discovered incidentally on sonography, appearing as a cystic lesion adjacent to or arising from the uterus, with walls comprising myometrium and a cavity communicating with the uterine lumen [10]. Primary UD is an extremely rare anomaly resulting from the failed midline fusion of the Müllerian duct during final uterine development. Weak points in the uterine wall may dilate during pregnancy and labor, forming a diverticulum [11]. Primary UD typically presents with symptoms including abnormal uterine bleeding and dysmenorrhea. Secondary UD is an iatrogenic condition that develops after uterine intervention or trauma. It may lead to abnormal placental attachment disorders, such as placenta accreta spectrum or ectopic pregnancy [12]. Ultrasound screening for the thinning of the uterine segment is crucial; however, this sign was not present in our case. UD can be misdiagnosed as degenerating uterine myoma, adenomyotic cyst, uterine malformations (such as unicornuate uterus, bicornuate uterus with single cervix, and incomplete septate uterus), or adnexal cyst based on sonographic appearance [13,14,15,16]. Another differential diagnosis is CI, defined as cervical length < 25 mm before 24 weeks’ gestation on transvaginal ultrasound, which provides an accurate measurement of the maximum closed cervical canal length [17]. Cervical funneling, characterized by a dilated endocervical canal with protruding fetal membranes, fetal parts, or umbilical cord, is a more reliable indicator [18]. Fundal pressure during transvaginal sonography may aid early CI detection in symptomatic women [19].
